# A survey of oonopid spiders in Taiwan with descriptions of three new species

**DOI:** 10.3897/zookeys.396.7033

**Published:** 2014-04-02

**Authors:** Yanfeng Tong, Shuqiang Li

**Affiliations:** 1 Chemistry and Life Science College, Shenyang Normal University, Shenyang 110034, China; 2 Institute of Zoology, Chinese Academy of Sciences, Beijing 100101, China

**Keywords:** Taxonomy, haplogyne, diagnosis, litter, island

## Abstract

The oonopid spiders of Taiwan are surveyed. Seven genera and 13 species are recognized, including 3 new species: *Ischnothyreus kentingensis* **sp. n.**, *Xyphinus hwangi* **sp. n.** and *Xestaspis shoushanensis* **sp. n.** Seven species are newly recorded from this region: *Brignolia parumpunctata* (Simon, 1893), *Opopaea apicalis* s (Simon, 1893), *Opopaea cornuta* Yin & Wang, 1984, *Opopaea deserticola* Simon, 1891, *Orchestina sinensis* Xu, 1987, *Pseudotriaeris karschi* (Bösenberg & Strand, 1906) and *Xestaspis loricata* (L. Koch, 1873).

## Introduction

Goblin spiders are small (1–4 mm), haplogyne, litter or canopy-dwelling, free hunting spiders. They are distributed nearly worldwide and are abundant in the tropics. Currently, the family includes 1325 described species in 97 genera (Platnick 2014).

The island of Taiwan is situated some 180 km off the southeastern coast of mainland China, and has an area of 35,883 km^2^. The oonopid spider fauna of this region has been poorly studied. To date, only four species, *Gamasomorpha cataphracta* Karsch, 1881, *Ischnothyreus narutomii* (Nakatsudi, 1942), *Ischnothyreus peltifer* (Simon, 1891) and *Opopaea sauteri* Brignoli, 1974, have been recorded from Taiwan (Brignoli 1974; Saaristo 2001). Our survey of the oonopid spiders of Taiwan was carried out from June 25 to July 3, 2013. The present paper expands the oonopid diversity of Taiwan to 8 genera and 14 species, including 3 new to science (described here) and 7 already-described species which are recorded from Taiwan for the first time. All the specimens were collected by sifting leaf litter.

## Material and methods

The specimens were examined using a Leica M205C stereomicroscope. Details were studied with the use of an Olympus BX51 compound microscope. All illustrations were made using a drawing tube and inked on ink jet plotter paper. Photos were made with a Canon EOS 550D zoom digital camera (18 megapixels). Vulvae were cleared in lactic acid. Scanning electron microscope images (SEM) were taken with a Hitachi S-4800. Measurements were taken using an Olympus BX51 compound microscope and are in millimeters.

The following abbreviations are used in the text: ALE = anterior lateral eyes; PLE = posterior lateral eyes; PME = posterior median eyes.

All specimens are deposited in the Institute of Zoology, Chinese Academy of Sciences in Beijing (IZCAS) and Shenyang Normal University in Shenyang (SYNU).

## Taxonomy

### Family Oonopidae Simon, 1890

#### Genus *Brignolia* Dumitrescu & Georgescu, 1983

##### Brignolia parumpunctata

(Simon, 1893)

http://species-id.net/wiki/Brignolia_parumpunctata

Brignolia cubana : Dumitrescu and Georgescu 1983: 107, pl. 22; Saaristo 2001: 343, figs 139–141, 142A–B, 143, 144A–B, 145.
Brignolia parumpunctata : Platnick et al. 2011: 14, figs 1–94.

###### Material examined.

CHINA: *Taiwan*: Pingtung County, Kenting, seaside near the Howard Beach Resort, 21°56'27.00"N, 120°48'26.68"E, elevation ca. 34 m, 25–28 June 2013, S. Li & Y. Tong leg., 1 ♂, 1 ♀ (SYNU-13); 1 ♂, 1 ♀ (SYNU-31).

###### Comments.

This species has been well described by many authors (i.e., Dumitrescu and Georgescu (1983), Saaristo (2001)). According to Platnick et al. (2011), this species widely distributed in North America, South America, South Asia, Southeast Asia, Seychelles Islands, Aurstalia and some Islands in South and West Pacific.

###### Distribution.

Pantropical. Newly recorded from Taiwan.

#### Genus *Gamasomorpha* Karsch, 1881

##### Gamasomorpha cataphracta

Karsch, 1881

http://species-id.net/wiki/Gamasomorpha_cataphracta

Gamasomorpha cataphracta : Brignoli 1974: 74, figs 1–6.

###### Material examined.

Not examined.

###### Comments.

Brignoli (1974) recorded this species from Akau (old name of Pingtung County), Taiwan.

###### Distribution.

China, Japan, Korea, Philippines.

#### Genus *Ischnothyreus* Simon, 1893

##### Ischnothyreus kentingensis sp. n.

http://zoobank.org/4E4779BF-82D5-470A-AE0C-4D5383D5CB1B

http://species-id.net/wiki/Ischnothyreus_kentingensis

Figs 1 – 3

###### Material examined.

Holotype ♂ (IZCAS AR 27808): CHINA: *Taiwan*: Pingtung County, Kenting, hills near the Howard Beach Resort, 21°56'27.00"N, 120°48'26.68"E, elevation ca. 34 m, 27 June 2013, S. Li & Y. Tong leg. Paratypes: same data as holotype, 1 ♂, 2 ♀ (SYNU-20); same data as holotype, 1 ♂, 5 ♀ (SYNU-58); same data as holotype, 1 ♂, 1 ♀ (SYNU-21).

###### Etymology.

The specific name is taken from the type locality; adjective.

###### Diagnosis.

**Figure 1.**
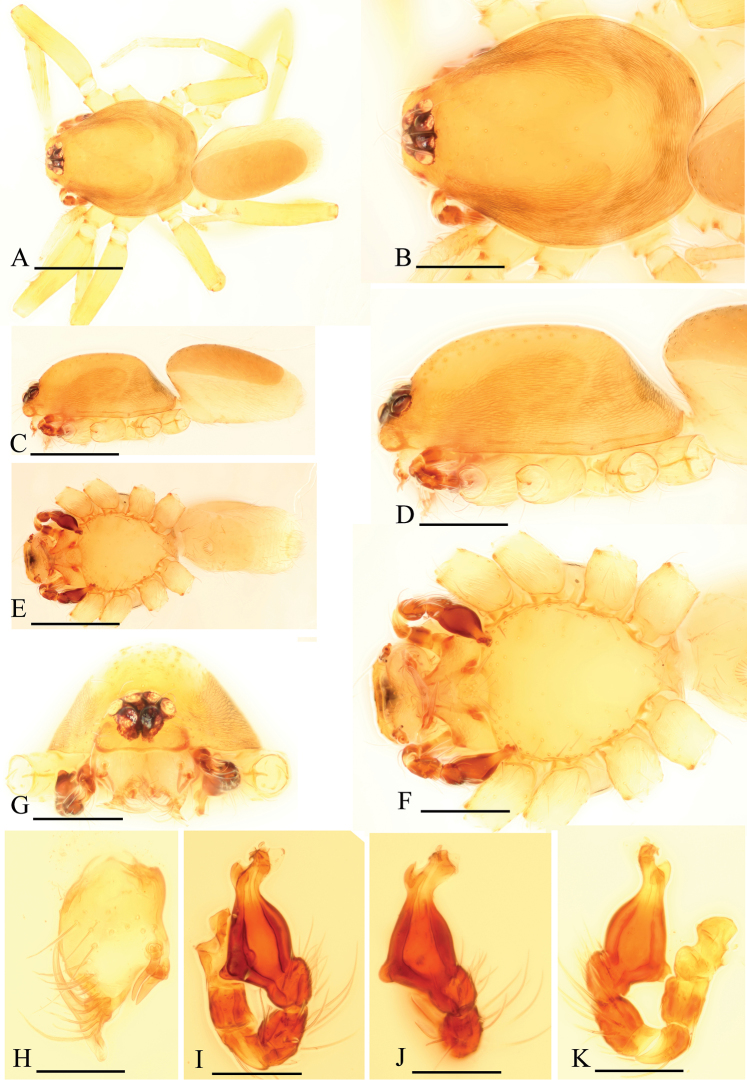
*Ischnothyreus kentingensis* sp. n., male. **A, C, E** habitus, dorsal, lateral and ventral views **B, D, F, G** prosoma, dorsal, lateral, ventral and anterior views **H** left chelicera, frontal view **I–K** left palp, retrolateral, dorsal and prolateral views. Scale bars: **A, C, E** = 0.4 mm; **B, D, F, G** = 0.2 mm; **H–K** = 0.1 mm.

**Figure 2.**
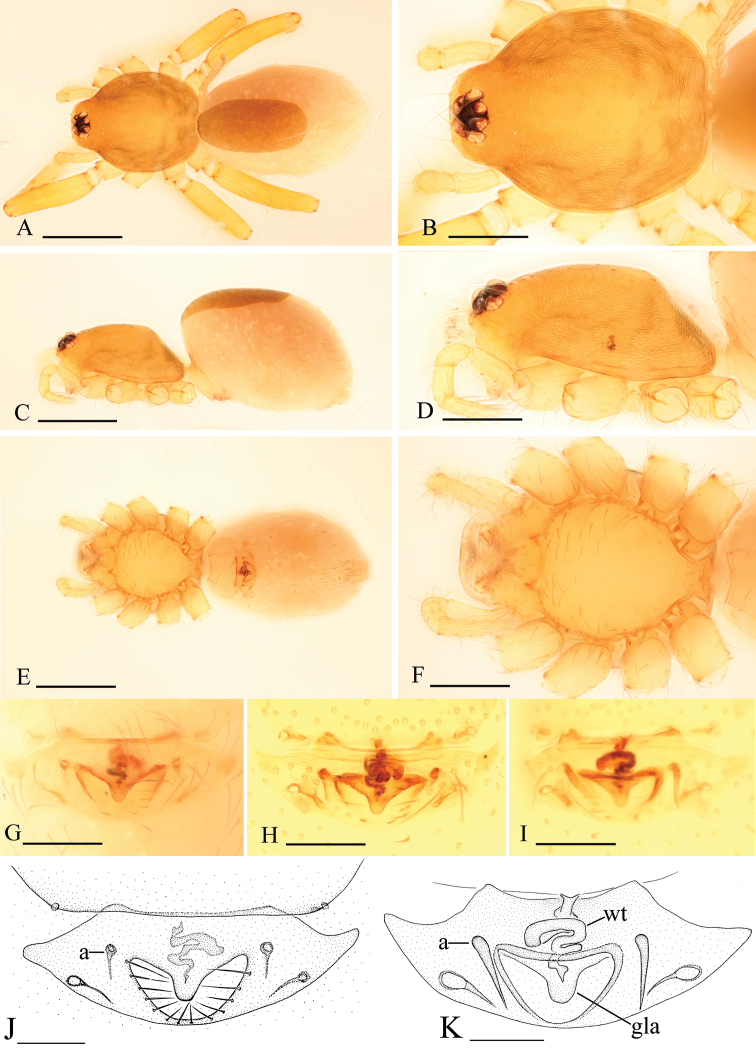
*Ischnothyreus kentingensis* sp. n., female. **A, C, E** habitus, dorsal, lateral and ventral views **B, D, F** prosoma, dorsal, lateral and ventral views **G, J** genital area, ventral view **H** genital area, ventral view (cleared in lactic acid) **I, K** genital area, dorsal view (cleared in lactic acid). Scale bars: **A, C, E** = 0.4 mm; **B, D, F** = 0.2 mm; **G–K** = 0.1 mm. Abbreviations: a = apodeme; gla = goblet-like atrium; wt = winding tube.

**Figure 3.**
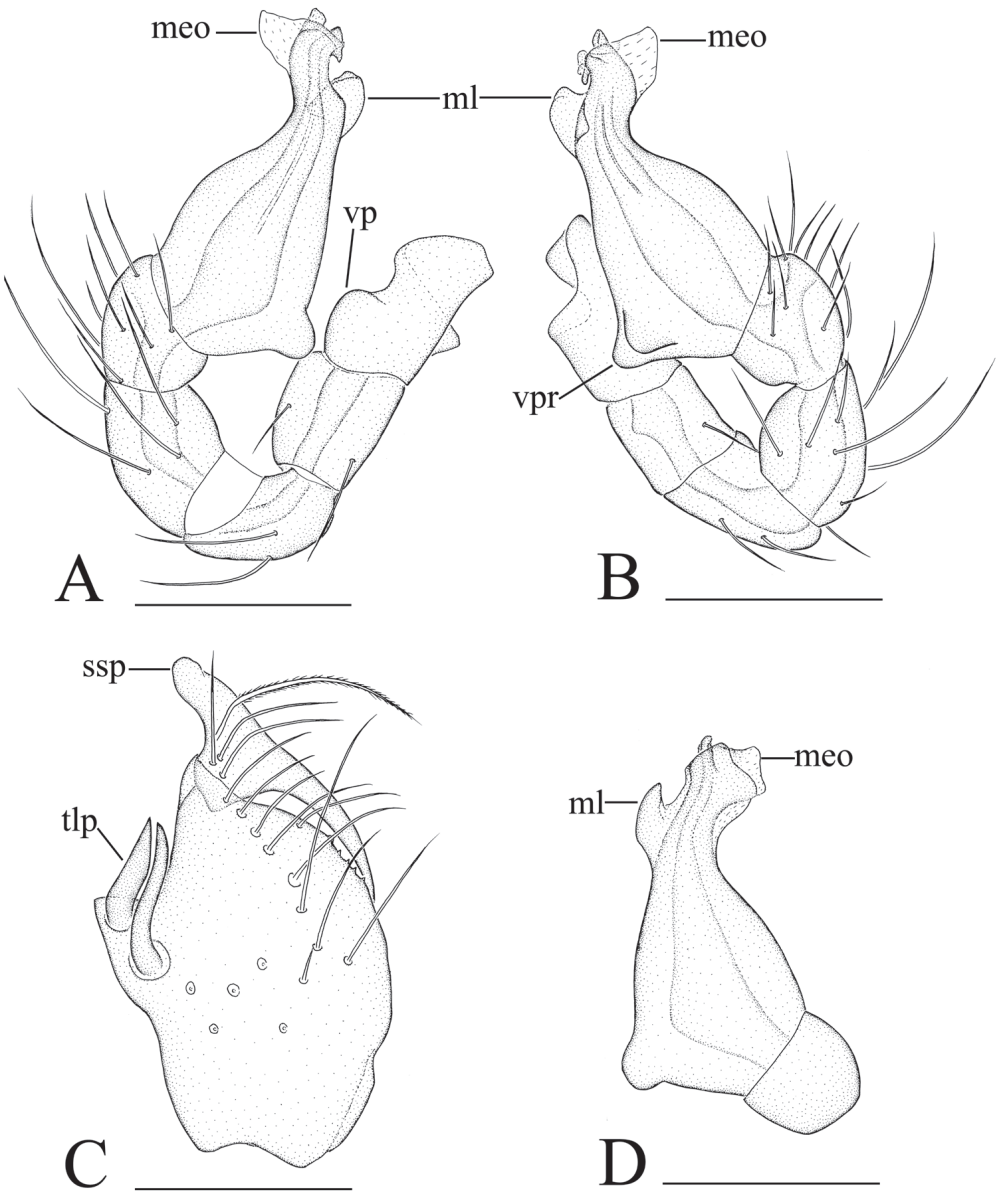
*Ischnothyreus kentingensis* sp. n., male. **A, B** left palp, prolateral and retrolateral views **C** left chelicera, frontal view **D** left palpal bulb, dorsal view. Scale bars: 0.1 mm. Abbreviations: meo = membranous outgrowth; ml = membranous lobe; ssp = slightly sclerotized process; tlp = thorn-like processes; vp = ventral projection; vpr = ventral protuberance.

The new species is similar to *Ischnothyreus spineus* Tong & Li, 2012, but can be distinguished by the male chelicerae which each bear two strong, short thorn-like processes (tlp in Figs 1H, 3C) and the female genital area possessing a large goblet-like atrium (Fig. 2G–K). The males of *Ischnothyreus spineus* bear only one long, curved thorn-like process on each of the chelicerae (see Tong and Li 2012: Figs 3H, 5C) and no visible atrium, with only a simple winding tube in female genital area (see Tong and Li 2012: Figs 4G, H, 5D, E).

###### Description.

Male (holotype). Total length 1.26; carapace 0.69 length, 0.54 width; abdomen 0.61 length, 0.33 width. Habitus as in Fig. 1A, C, E. *Carapace*: orange-brown, with brown egg-shaped patches behind eyes, oval in dorsal view, pars cephalica strongly elevated in lateral view, surface and sides strongly reticulate (Fig. 1B, D). *Eyes*: six, in one group, well developed, ALE largest, PME and PLE nearly equal sized; posterior eye row straight from above, procurved from front (Fig. 1G). *Mouthparts*: chelicerae slightly divergent, with a slightly sclerotized process at base of fangs (ssp) and two strong, thorn-like processes (tlp) in the middle of the retrolateral margin; fang groove with a few small denticles (Figs 1H, 3C). Anterior margin of labium not indented at middle. Anteromedian tip of endites with one strong, tooth-like projection (Fig. 1F). *Abdomen*: posterior spiracles not connected by groove. Pedicel tube short, unmodified, scutum extending far dorsal of pedicel. Dorsal scutum covering about 4/5 of abdomen, about equal to the abdomen width, not fused to epigastric scutum. Epigastric and postepigastric scutum well sclerotized, pale orange, fused. *Leg spine formula*: femur I with 2 prolateral and 1 small retrolateral spine, tibia I with 4 pairs, metatarsus I with 2 pairs of long ventral spines. Spination of leg II similar to leg I except femur with only one prolateral spine. Legs III and IV spineless. *Genitalia*: sperm pore situated at level of anterior spiracles. Palp strongly sclerotized, trochanter with ventral projection (vp); patella about as long as femur, not enlarged; cymbium brown, not fused with bulb, bulb brown, more than twice as long as cymbium, stout, tapering apically, with two small ventral protuberances (vpr), at the bending site with a membranous lobe (ml), distal part of bulb with membranous outgrowth (meo) (Figs 1I–K, 3A, B, D).

Female (paratype). Total length 1.51; carapace 0.64 length, 0.52 width; abdomen 0.87 length, 0.56 width. Habitus as in Fig. 2A, C, E. As in male except as noted. *Carapace*: without any pattern, pars cephalica slightly elevated in lateral view (Fig. 2B, D). *Mouthparts*: chelicerae and endites unmodified (Fig. 2F). *Abdomen*: dorsal scutum covering about 2/3 of abdomen, about 1/2 of abdomen width. Postepigastric scutum elongated hexagonal, not fused to epigastric scutum, with short posteriorly directed lateral apodemes (a) (Fig. 2G, H, J). *Genitalia*: at the middle of the anterior edge of the postepigastric scutum runs a dark, strongly winding tube posteriorly (wt), ending in a large goblet-like atrium (gla) close to posterior edge of scutum (Fig. 2I, K).

###### Distribution.

Known only from the type locality.

##### Ischnothyreus narutomii

(Nakatsudi, 1942)

http://species-id.net/wiki/Ischnothyreus_narutomii

Gamasomorpha narutomii : Nakatsudi 1942: 287, figs 1.1–6.
Ischnothyreus narutomii : Lee 1966: 22, fig. 4c–e; Tong and Li 2008: 60, fig. 5A–D; Ono 2009: 103, figs 18–24; Tong 2013: 32, fig. 47A–D.

###### Material examined.

CHINA: *Taiwan*: Nantou County, Ren’ai Town, Songgang Village, 24°05'13.30"N, 121°10'20.07"E, elevation ca. 2067 m, 2 July 2013, S. Li, G. Zheng & Y. Tong leg., 1 ♂, 1 ♀ (SYNU-23); 4 ♂, 5 ♀ (SYNU-24); 5 ♂, 10 ♀ (SYNU-49).

###### Comments.

Lee (1966) recorded this species in Taichung City of Taiwan; this species has also been found in the Hainan Province of China and in Japan.

###### Distribution.

China, Japan.

##### Ischnothyreus peltifer

(Simon, 1891)

http://species-id.net/wiki/Ischnothyreus_peltifer

Ischnothyreus peltifer : Saaristo 2001: 345, figs 146A, 147A–C, 148A–B, 149–150, 152–153, 154,155A, 156, 157A–B; Saaristo and van Harten 2006: 135, figs 15a–b, 16a–c, 17.

###### Material examined.

CHINA: *Taiwan*: Pingtung County, Kenting, seaside near Howard Beach Resort, 21°56'27.00"N, 120°48'26.68"E, elevation ca. 34 m, 25–28 June 2013, S. Li & Y. Tong leg., 1 ♀ (SYNU-22); 3 ♀ (SYNU-52).

###### Comments.

Brignoli (1974) described *Ischnothyreus formosus* from Akau (old name of Pingtung County), Taiwan. Saaristo (2001) synonymized this species with *Ischnothyreus peltifer* and considered it to be a widely-distributed species in the tropics.

###### Distribution.

Pantropical, Canada and Europe (introduced).

#### Genus *Opopaea* Simon, 1891

##### Opopaea apicalis

s (Simon, 1893)

http://species-id.net/wiki/Opopaea_apicalis

Epectris apicalis : Simon 1893: 74; Platnick and Dupérré 2009: 30, figs 105–160.
Opopaea lena : Saaristo 2001: 337, figs 112A–C, 113–117.
Opopaea apicalis : Baehr et al. 2013: 109.

###### Material examined.

CHINA: *Taiwan*: Pingtung County, Kenting, seaside near Howard Beach Resort, 21°56'27.00"N, 120°48'26.68"E, elevation ca. 34 m, 25–28 June 2013, S. Li & Y. Tong leg., 3 ♂, 3 ♀ (SYNU-12); 8 ♂, 14 ♀ (SYNU-33).

###### Comments.

This species has been well described by Platnick and Dupérré (2009).

###### Distribution.

Pantropical. Newly recorded from Taiwan.

##### Opopaea cornuta

Yin & Wang, 1984

http://species-id.net/wiki/Opopaea_cornuta

Opopaea cornuta : Yin and Wang 1984: 52, fig. 2A–F; Tong and Li 2010: 24, figs 1B, K, 2A–E, 9A–F; Tong 2013: 37, figs 25B, K, 53A–E, 54A–F.

###### Material examined.

CHINA: *Taiwan*: Nantou County, Huisun Forest Area, 24°05'16.74"N, 121°02'04.69"E, elevation ca. 788 m, 30 June to 1 July 2013, S. Li, G. Zheng & Y. Tong leg., 1 ♀ (SYNU-44).

###### Comments.

Yin and Wang (1984) reported this species from Hunan and Yunnan Provinces. Tong and Li (2008) redescribed this species from Hainan Province. This species seems to be widely distributed in southern China and the adjacent areas.

###### Distribution.

China, Laos. Newly recorded from Taiwan.

##### Opopaea deserticola

Simon, 1891

http://species-id.net/wiki/Opopaea_deserticola

Opopaea deserticola : Simon 1891: 560, pl. 42, fig. 5; Saaristo 2001: 333, figs 93A–98A, 99–101; Platnick and Dupérré 2009: 4, figs 1–72; Tong and Li 2010: 35, figs 1Q, S–T, 7A–C; Tong 2013: 37, fig. 25Q, S–T.

###### Material examined.

CHINA: *Taiwan*: Kaohsiung City, Shoushan Mountain, 29 June 2013, S. Li, G. Zheng & Y. Tong leg., 4 ♂, 4 ♀ (SYNU-14); Pingtung County, Kenting, hills near Howard Beach Resort, 27 June 2013, S. Li & Y. Tong leg., 1 ♂, 1 ♀ (SYNU-15); Nantou County, Huisun Forest Area, 24°05'16.74"N, 121°02'04.69"E, elevation ca. 788 m, 30 June to 1 July 2013, S. Li, G. Zheng & Y. Tong leg., 6 ♂, 6 ♀ (SYNU-16); 15 ♂, 21 ♀ (SYNU-28); 18 ♂, 22 ♀ (SYNU-48); Pingtung County, Kenting, Sheding Nature Park, 21°57'25.15"N, 120°49'06.29"E, elevation ca. 221 m, 26 June 2013, S. Li & Y. Tong leg., 1 ♂, 2 ♀ (SYNU-50).

###### Comments.

This species has been well described by Platnick and Dupérré (2009).

###### Distribution.

Pantropical. Newly recorded from Taiwan.

##### Opopaea sauteri

Brignoli, 1974

http://species-id.net/wiki/Opopaea_sauteri

Opopaea sauteri : Brignoli 1974: 82, figs 19–21; Tong and Li 2010: 35, figs 1G, N, P, R, U, 6A–G; Tong 2013: 42, figs 25G, N, P, R, U, 61A–G.

###### Material examined.

CHINA: *Taiwan*: Pingtung County, Kenting, seaside near Howard Beach Resort, 21°56'27.00"N, 120°48'26.68"E, elevation ca. 34 m, 25–28 June 2013, S. Li & Y. Tong leg., 1 ♂, 1 ♀ (SYNU-17); 2 ♀ (SYNU-32).

###### Comments.

Brignoli (1974) described this species from Takao (old name of Kaohsiung City), Taiwan. Tong and Li (2010) redescribed this species from Hainan Province of China.

###### Distribution.

China.

#### Genus *Orchestina* Simon, 1882

##### Orchestina sinensis

Xu, 1987

http://species-id.net/wiki/Orchestina_sinensis

Orchestina sinensis : Xu 1987: 256, figs 1–6.

###### Material examined.

CHINA: *Taiwan*: Pingtung County, Kenting, hills near Howard Beach Resort, 27 June 2013, S. Li & Y. Tong leg., 1 ♀ (SYNU-40); Nantou County, Huisun Forest Area, 24°05'16.74"N, 121°02'04.69"E, elevation ca. 788 m, 30 June to 1 July 2013, S. Li, G. Zheng & Y. Tong leg., 2 ♀ (SYNU-45); Kaohsiung City, Shoushan Mountain, 29 June 2013, S. Li, G. Zheng & Y. Tong leg., 1 ♂ (SYNU-53).

###### Comments.

This species has been recorded from the Anhui and Zhejiang Provinces of China. It seems to be widely distributed in southern China.

###### Distribution.

Southern China. Newly recorded from Taiwan.

#### Genus *Pseudotriaeris* Brignoli, 1974

##### Pseudotriaeris karschi

(Bösenberg & Strand, 1906)

http://species-id.net/wiki/Pseudotriaeris_karschi

Gamasomorpha karschi : Bösenberg and Strand 1906: 117, pl. 16, fig. 455.
Pseudotriaeris karschi : Brignoli 1974: 77, figs 7–11; Song 1987: 96, fig. 60.
Pseudotriaeris echinatus : Yin and Wang 1984: 55, fig. 4A–K.

###### Material examined.

CHINA: *Taiwan*: Pingtung County, Kenting, seaside near Howard Beach Resort, 21°56'27.00"N, 120°48'26.68"E, elevation ca. 34 m, 25–28 June 2013, S. Li & Y. Tong leg., 2 ♂, 2 ♀ (SYNU-19); 4 ♂, 2 ♀ (SYNU-41).

###### Comments.

Brignoli erected the genus *Pseudotriaeris* in 1974, based on the type species *Pseudotriaeris karschi* from Japan. Yin and Wang (1984) described *Pseudotriaeris echinatus* from Hunan, China, but it was synonymized with the type species by Song (1987). Currently, this species is known from Anhui, Hunan and Zhejiang Provinces of China and from Japan. However, the species *Pseudotriaeris karschi* has never been studied in detail. The specimens from China have not been compared with the type specimens, and may belong to one or more different species. The generic characters, such as the male palps with complicated apophyses and the male endites with a backwards folded ridge, are very similar to those of the genus *Xyphinus* Simon, 1893 (see Deeleman-Reinhold 1987). We suspect that this genus can be synonymized with *Xyphinus*. A thorough investigation of the type species *Pseudotriaeris karschi* is now required.

###### Distribution.

China, Japan. Newly recorded from Taiwan.

#### Genus *Xyphinus* Simon, 1893

##### Xyphinus hwangi sp. n.

http://zoobank.org/DCCD5A23-F3B9-44F5-A361-1D8499B77D09

http://species-id.net/wiki/Xyphinus_hwangi

**Figure 4.**
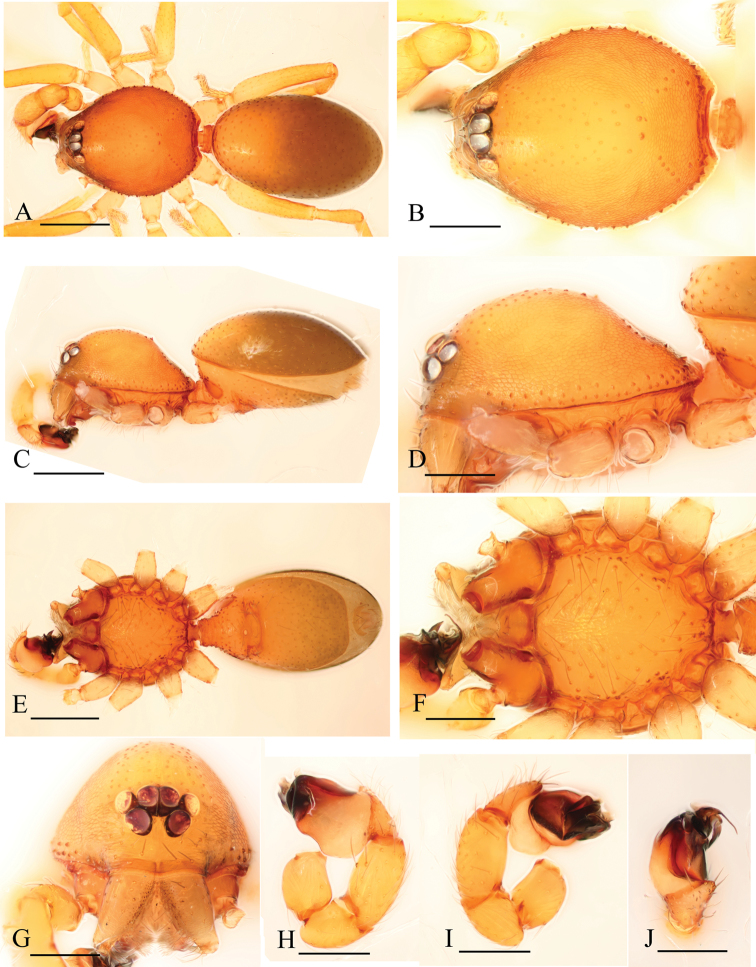
*Xyphinus hwangi* sp. n., male. **A, C, E** habitus, dorsal, lateral and ventral views **B, D, F, G** prosoma, dorsal, lateral, ventral and anterior views **H–J** left palp, retrolateral, prolateral and dorsal views. Scale bars: **A, C, E** = 0.4 mm; **B, D, F–J**= 0.2 mm.

Figs 4 [Fig F5] [Fig F6] –7

###### Material examined.

Holotype ♂ (IZCAS AR 27809): CHINA: *Taiwan*: Kaohsiung City, Shoushan Mountain, 29 June 2013, S. Li, G. Zheng & Y. Tong leg. Paratypes: same data as holotype, 3 ♂, 5 ♀ (SYNU-18); same data as holotype, 12 ♂, 23 ♀ (SYNU-36); same data as holotype, 2 ♂, 1 ♀ (SYNU-47).

###### Etymology.

The specific name is a patronym honoring Dr. Chung-Chi Hwang (National University of Kaohsiung), who is a leading taxonomist of terrestrial snails in Taiwan.

###### Diagnosis.

The new species is similar to *Pseudotriaeris karschi* (see Brignoli 1974), but can be distinguished by the long, slender and strongly curved ventral apophysis (va in Fig. 6F) in the male palp and the large nose-shaped protuberance (nos in Figs 6L, 7D) in the female epigastric area.

###### Description.

Male (holotype). Total length 1.87; carapace 0.81 length, 0.63 width; abdomen 0.99 length, 0.61 width. Habitus as in Fig. 4A, C, E. *Carapace*: orange, dorsal scutum yellow-brown, chelicerae, sternum, legs and ventral scutum light yellow. Carapace dorsally smooth, covered with rows of short hairs; sides finely reticulate; carapace margin with two rows of small denticles on either side and some larger denticles on the posterior slope (Fig. 4B, D). No fovea. Posterior pits lacking. Eyes six, ALE largest, PLE smallest; posterior eye row slightly recurved from above, straight from front. Clypeus with sinuous anterior margin; clypeus height about 1.5 times the diameter of anterior eyes (Figs 4G, 6A). *Mouthparts*: chelicerae toothless, with many small granules on the promargin (Fig. 6B, C, M). Endites with backwards folded ridge. Sternum smooth (Fig. 4F). Legs spineless. *Abdomen*: shape of abdomen normal, not overlapping the carapace. Dorsal scutum ovoid, smooth, nearly entirely covering the abdomen. Booklung covers ovoid, large. Pedicel tube ribbed. Scuto-pedicel region unmodified. Posterior spiracles connected by groove. Postepigastric scutum strongly sclerotized; spinneret scutum present as an incomplete ring. *Genitalia*: sperm pore oval, medium sized, situated at level of anterior spiracles. Palp (Figs 4H–J, 6D–I, 7A–C): femur inserted near the middle of patella; patella about as long as femur; cymbium strongly protruding prolaterally; bulb with complicated apophyses, ventral apophysis very slender and strongly curved (va in Fig. 6F).

**Figure 5. F5:**
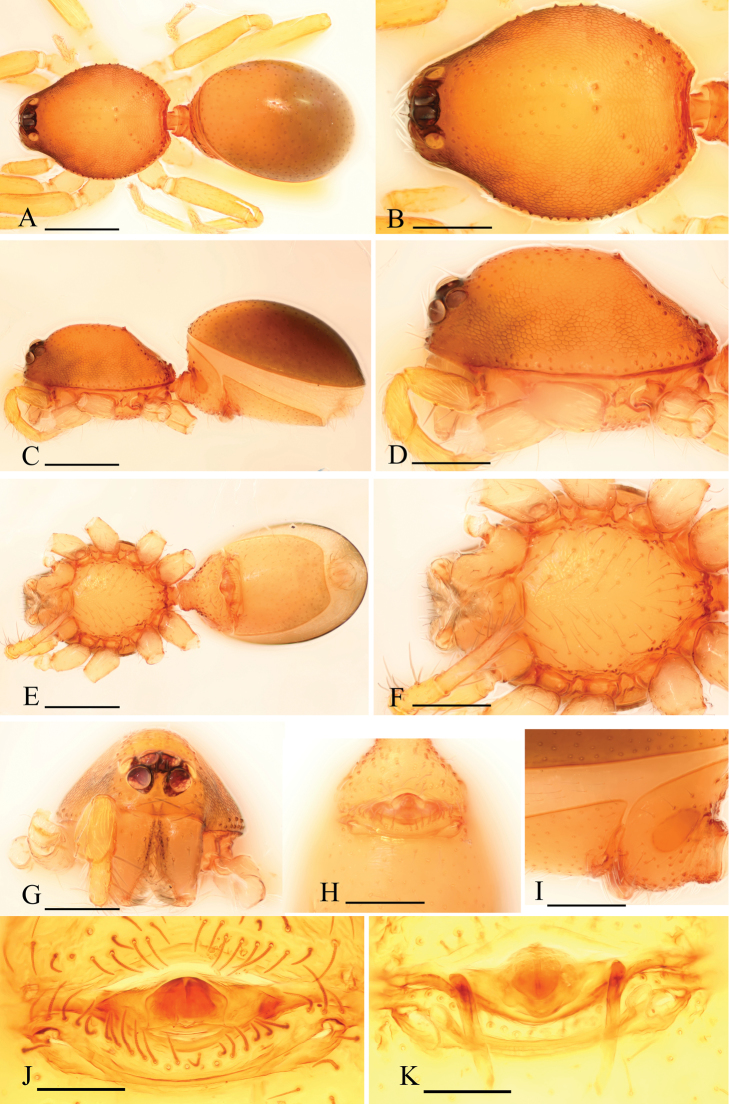
*Xyphinus hwangi* sp. n., female. **A, C, E** habitus, dorsal, lateral and ventral views **B, D, F, G** prosoma, dorsal, lateral, ventral and anterior views **H, I** abdomen, ventral and lateral views **J, K** genital area, ventral and dorsal views (cleared in lactic acid). Scale bars: **A, C, E** = 0.4 mm; **B, D, F–I** = 0.2 mm; **J, K** = 0.1 mm.

**Figure 6. F6:**
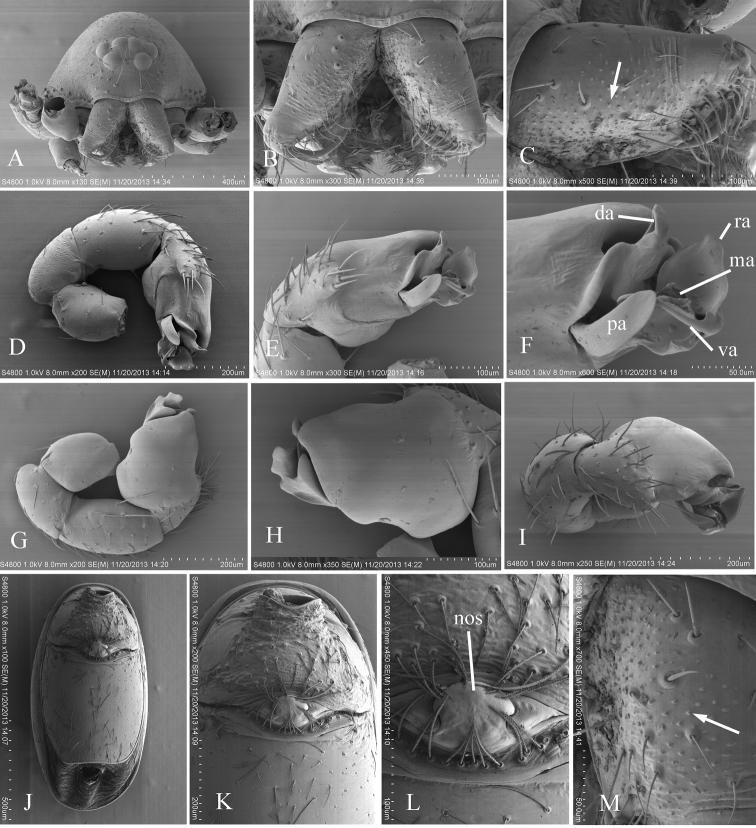
*Xyphinus hwangi* sp. n., SEM. **A** male prosoma, anterior view **B, C, M** male chelicerae, frontal view (arrow shows the small granules) **D, G, I** male left palp, prolateral, retrolateral and dorsal views **E, H** male left palpal bulb, prolateral and retrolateral views **F** distal part of male left palpal bulb, prolateral view **J** female abdomen, ventral view **K, L** female genital area, ventral view. Abbreviations: da = dorsal apophysis; ma = medial apophysis; nos = nose-shaped protuberance; pa = prolateral apophysis; ra = retrolateral apophysis; va = ventral apophysis.

**Figure 7.**
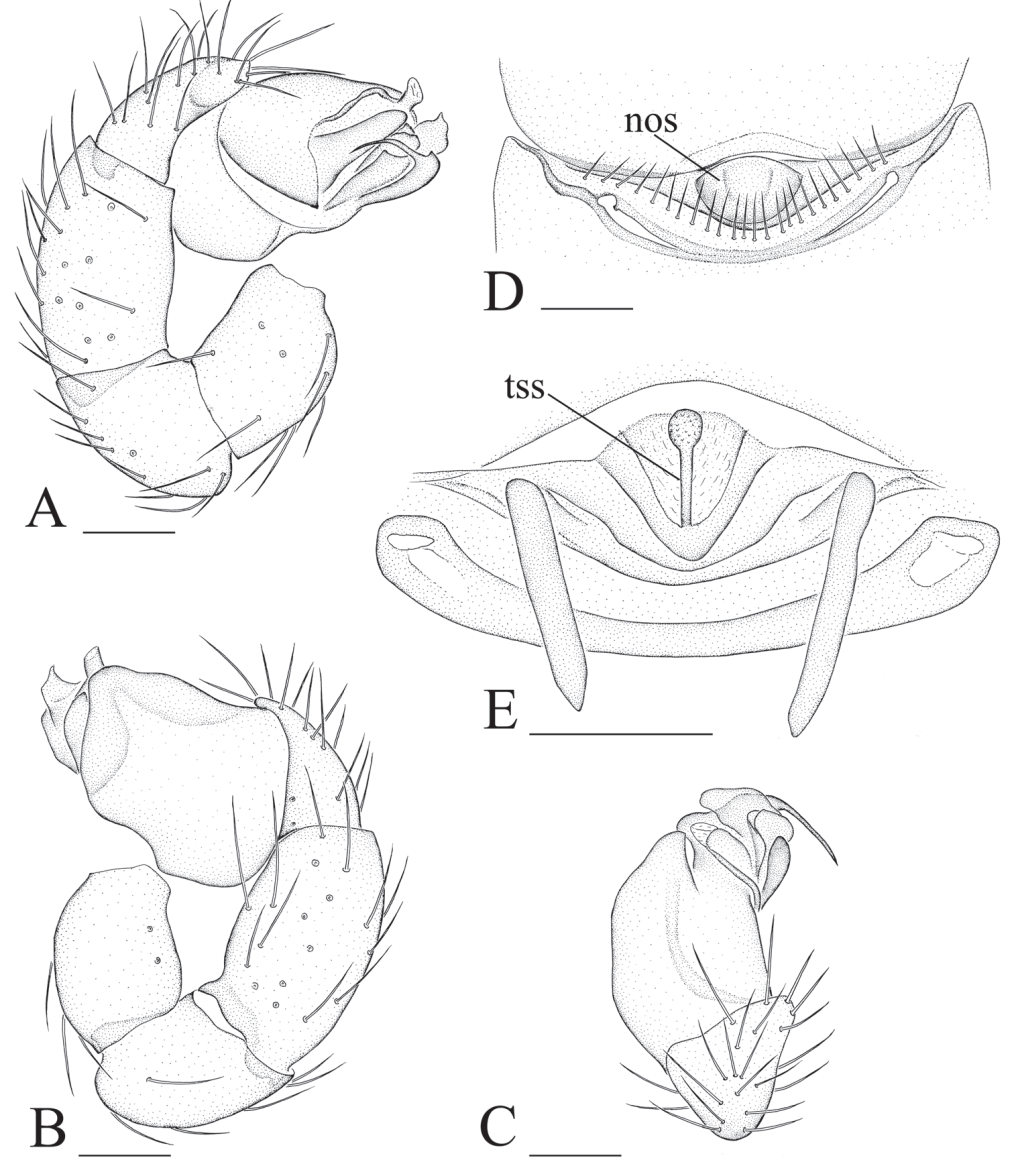
*Xyphinus hwangi* sp. n. **A–C** male left palp, prolateral, retrolateral and dorsal views **D, E** female genital area, ventral and dorsal views. Scale bar: 0.1 mm. Abbreviations: nos = nose-shaped protuberance; tss = thin stick-shape sclerite.

Female (paratype). Total length 1.84; carapace 0.81 length, 0.62 width; abdomen 0.95 length, 0.92 width. Habitus as in Fig. 5A, C, E. As in male except as noted. Endites unmodified (Fig. 5F). Postepigastric scutum rectangular, not fused to epigastric scutum, with long posteriorly directed lateral apodemes. *Genitalia*: with a large nose-shaped protuberance (nos in Figs 6L, 7D) at the middle of the anterior edge of the postepigastric scutum; in dorsal view, a thin stick-shape sclerite extending anteriorly (tss in Figs 5K, 7E).

###### Distribution.

Known only from the type locality.

#### Genus *Xestaspis* Simon, 1884

##### Xestaspis loricata

(L. Koch, 1873)

http://species-id.net/wiki/Xestaspis_loricata

Gamasomorpha loricata : Saaristo 2001: 311, figs 1B, 2B, 3B.
Xestaspis loricata : Tong and Li 2009: 26, figs 1G–I, 2G–I, 5A–D; Tong 2013: 51, figs 17G–I, 18G–I, 69A–D.

###### Material examined.

CHINA: *Taiwan*: Pingtung County, Kenting, seaside near Howard Beach Resort, 21°56'27.00"N, 120°48'26.68"E, elevation ca. 34 m, 25–28 June 2013, S. Li & Y. Tong leg., 2 ♀ (SYNU-30); Nantou County, Huisun Forest Area, 24°05'16.74"N, 121°02'04.69"E, elevation ca. 788 m, 30 June to 1 July 2013, S. Li, G. Zheng & Y. Tong leg., 2 ♀ (SYNU-43).

###### Comments.

Well described in above mentioned papers.

###### Distribution.

Australia, China, Laos, Micronesia. Newly recorded from Taiwan.

##### Xestaspis shoushanensis sp. n.

http://zoobank.org/D9171ACC-20CA-437F-8836-A9D5CCA61EA3

http://species-id.net/wiki/Xestaspis_shoushanensis

Figs 8 – 10

###### Material examined.

Holotype ♂ (IZCAS AR 27810): CHINA: *Taiwan*: Kaohsiung City, Shoushan Mountain, 29 June 2013, S. Li, G. Zheng & Y. Tong leg. Paratypes: same data as holotype, 1 ♀ (SYNU-11); same data as holotype, 2 ♀ (SYNU-57).

###### Etymology.

The specific name is taken from the type locality; adjective.

###### Diagnosis.

**Figure 8.**
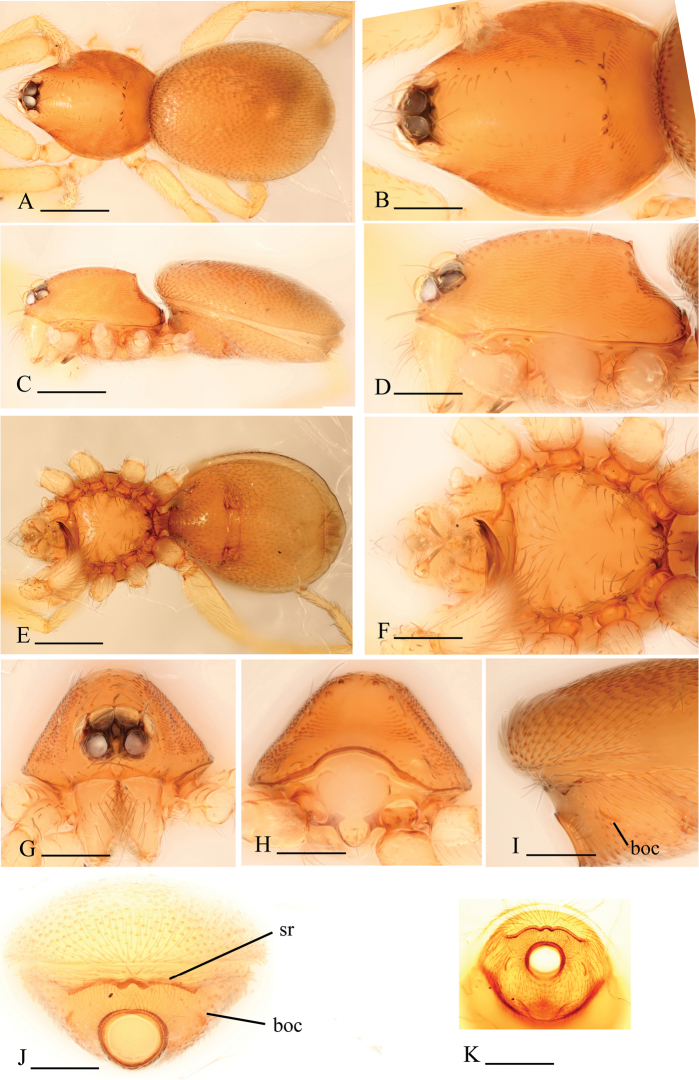
*Xestaspis shoushanensis* sp. n., male. **A, C, E** habitus, dorsal, lateral and ventral views **B, D, F, G, H** prosoma, dorsal, lateral, ventral, anterior and posterior views **I** booklung covers, lateral view **J, K** abdomen, anterior and anteroventral views. Scale bars: **A, C, E, K** = 0.4 mm; **B, D, F–J** = 0.2 mm. Abbreviations: boc = booklung covers; sr = scutal ridge.

**Figure 9.**
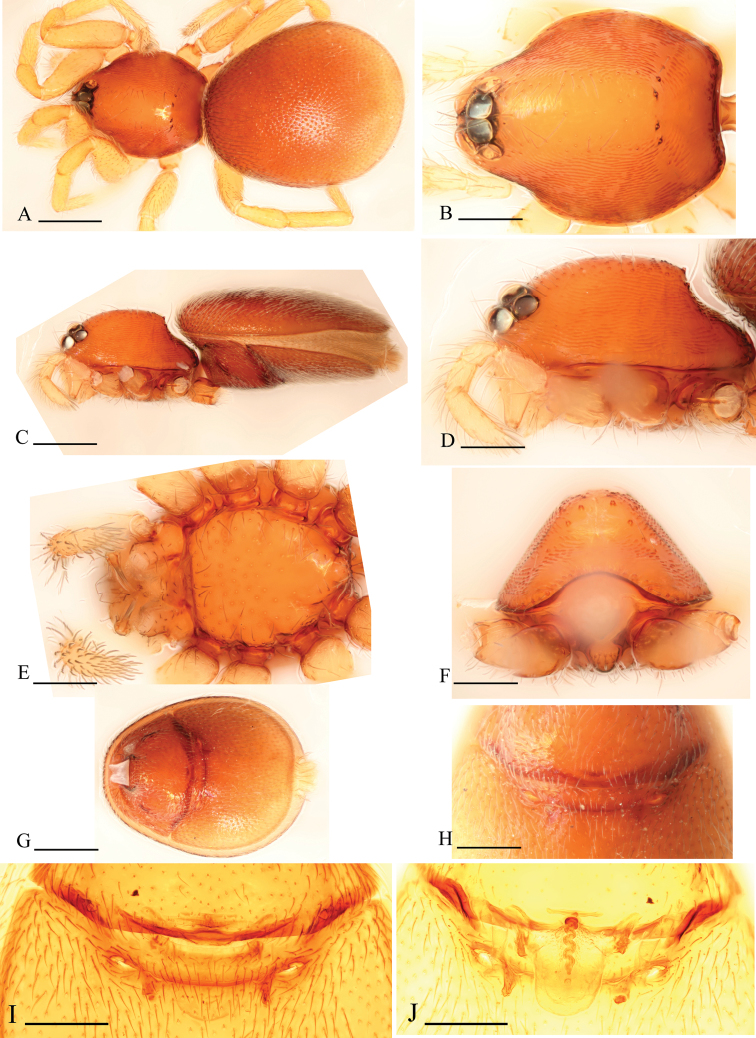
*Xestaspis shoushanensis* sp. n., female. **A, C** habitus, dorsal and lateral views **B, D, E, F** prosoma, dorsal, lateral, ventral and posterior views **G** abdomen, ventral view **H** genital area, ventral view **I, J** genital area, ventral and dorsal views (cleared in lactic acid). Scale bars: **A, C, G** = 0.4 mm; **B, D–F, H–J** = 0.2 mm.

**Figure 10.**
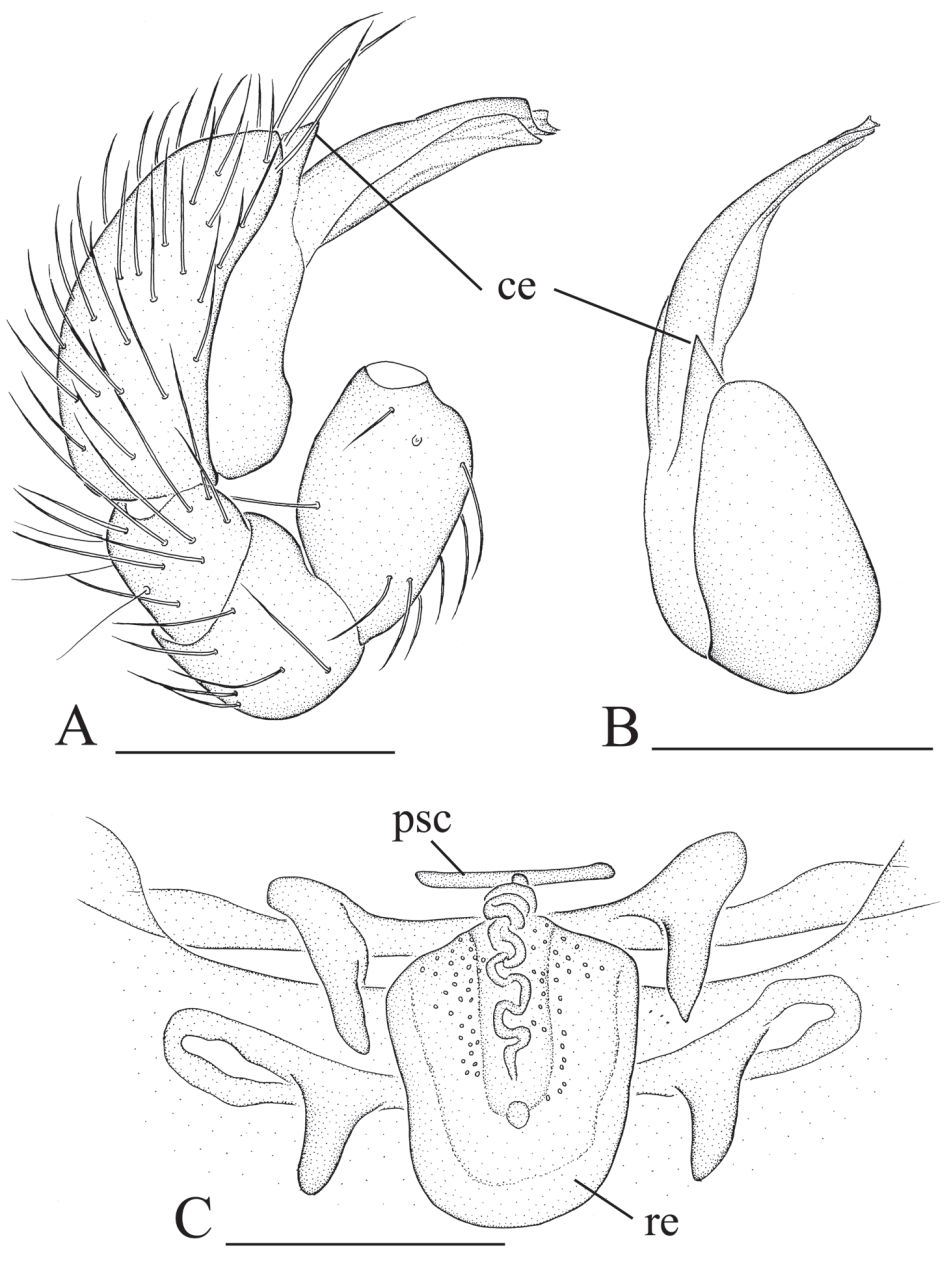
*Xestaspis shoushanensis* sp. n. **A** male left palp, prolateral view **B** male left palpal bulb, dorsal view **C** genital area, dorsal view. Scale bar: 0.1 mm. Abbreviations: ce = conical extension; psc = paddle-like sclerite; re = receptaculum.

The new species is similar to *Xestaspis paulina* (see Eichenberger et al. 2012), but can be distinguished by the sternum with short radial furrows between coxae I–II, II–III and III–IV (Figs 8F, 9E), by the lateral carapace surface, which is strongly striated (Figs 8B, D, 9B, D), by the abdominal scuto-pedicel region with only one straight scutal ridge, without a second, upper, semicircular ridge (Fig. 8J, K), and by the male palp with strongly pointed conical extension (ce in Fig. 10A, B).

###### Description.

Male (holotype). Total length 1.87; carapace 0.82 length, 0.65 width; abdomen 1.09 length, 0.84 width. Habitus as in Fig. 8A, C, E. Body yellow or reddish brown, chelicerae and sternum lighter, legs yellow. *Carapace*: pars cephalica slightly elevated in lateral view. Carapace dorsally smooth, with two rows of short, finely hairs laterally; sides strongly striated; lateral margin with a row of finely hairs. All eyes oval, about subequal; clypeus height about equal to the diameter of anterior eyes (Fig. 8B, D, G). Sternum with narrow, transverse palpal groove, covered with thin hairs standing in small pits, radial furrows present (Fig. 8F). *Abdomen*: dorsal scutum ovoid, punctate, densely covered with short hairs. Booklung covers very small, anterolateral edge with tubercle. Pedicel tube short, without dorsolateral extension, scuto-pedicellar region with straight scutal ridge (Fig. 8J, K). Colulus very small, bearing two setae. *Genitalia*: sperm pore narrow, slit-like. Palp (Fig. 10A, B): cymbium and bulbus yellow. Bulbus distally tapering, ending as pointed conical extension (ce). Cymbium not extending beyond distal tip of bulb. Embolus-conductor complex, mesially bent in dorsal view.

Female (paratype). Total length 2.11; carapace 0.86 length, 0.69 width; abdomen 1.28 length, 1.05 width. Habitus as in Fig. 9A, C. As in male except as noted. *Genitalia*: ventral view: simple, externally without special features (Fig. 9H, I). Dorsal view: vulva with a small receptaculum and complicated sclerites (Figs 9J, 10C).

###### Distribution.

Known only from the type locality.

## Supplementary Material

XML Treatment for Brignolia parumpunctata

XML Treatment for Gamasomorpha cataphracta

XML Treatment for Ischnothyreus kentingensis

XML Treatment for Ischnothyreus narutomii

XML Treatment for Ischnothyreus peltifer

XML Treatment for Opopaea apicalis

XML Treatment for Opopaea cornuta

XML Treatment for Opopaea deserticola

XML Treatment for Opopaea sauteri

XML Treatment for Orchestina sinensis

XML Treatment for Pseudotriaeris karschi

XML Treatment for Xyphinus hwangi

XML Treatment for Xestaspis loricata

XML Treatment for Xestaspis shoushanensis
